# Look before you leap - individual variation in social vigilance shapes socio-spatial group properties in an agent-based model

**DOI:** 10.1007/s00265-012-1342-3

**Published:** 2012-03-14

**Authors:** Ellen Evers, Han de Vries, Berry M. Spruijt, Elisabeth H. M. Sterck

**Affiliations:** 1 Behavioural Biology, Utrecht University, Padualaan 8, 3584CH Utrecht, the Netherlands; 2 Ethology Research, Biomedical Primate Research Center, Lange Kleiweg 161, 2288GJ Rijswijk, the Netherlands

**Keywords:** Social vigilance, Social attention, Aggression, Social behavior, Individual-based model, Self-organization

## Abstract

Next to predator detection, primate vigilance also serves to keep track of relevant conspecifics. The degree of vigilance towards group members often reflects the dominance rank of an individual: subordinates pay attention to dominants. Although it has been suggested that subordinates’ vigilance may result in spatial centrality of dominants, this has not been addressed in either empirical or modeling studies. Using agent-based models, we determined how social vigilance affects socio-spatial properties of primate groups. A basic model without social vigilance, where individuals avoid potential aggressors (*avoidance model*), was contrasted with two models that each additionally included a different type of social vigilance: a) monitoring a specific potential aggressor to remain informed on its whereabouts (*monitoring model*) or b) scanning the whole group to detect potential aggressors (*scanning model*). Adding monitoring or scanning behavior to the *avoidance model* reinforced spatial centrality of dominants, a pattern often observed in primates, and resulted in more spread out groups. Moreover, variation in scanning tendency alone was already sufficient to generate spatial centrality of dominants: frequently scanning subordinates could move further away from the group center than dominants, before losing sight of group members. In the *monitoring model*, two mechanisms caused decreased encounter frequencies among subordinates: a) increased inter-individual distances, and b) frequent monitoring of central dominants. In the *scanning model*, encounters among subordinates decreased due to increased inter-individual distances. This agent-based model study provides a clear indication that individual variation in social vigilance may be an important structuring feature of primate social groups.

## Introduction

Group-living animals can afford to spend less time on vigilance behavior towards potential predators than solitary animals (the “many eyes effect”: Pulliam 1973; Powell 1974). However, time spent on vigilance actually increases with group size in many group-living primates (Elgar 1989; Roberts 1996), revealing that vigilance behavior may not only be directed at external threats, but also at conspecifics (Treves 2000; Hirsch 2002). Primates use social vigilance, or “social attention”, to track the whereabouts and the behavior of relevant conspecifics, such as offspring, potential mates or potentially aggressive group members (Keverne et al. 1978; Altmann 1980; Caine and Marra 1988; Maestripieri 1993; Watts 1998; Cowlishaw 1998; Kutsukake 2006). Information gained by social vigilance may affect social and spatial group patterns. In this paper we use agent-based models to study the effect of employing social vigilance on spatial group patterns and the distribution of encounters among group members.

More than 40 years ago, Chance (1956) and Chance and Jolly (1970) proposed the importance of a “social attention structure”, a property of the whole group which is evident in who attends to whom (Immelmann and Beer 1989; Barrows 2001). More recently, researchers have found that many primate species show individual variation in the direction and frequency of social vigilance. Often, social vigilance is employed more frequently by subordinates than by dominants and is directed up the dominance hierarchy, e.g. in macaques (Haude et al. 1976; Deaner et al. 2005), baboons (Alberts 1994), capuchins (Pannozzo et al. 2007) patas monkeys (McNelis and Boatright-Horowitz 1998), talapoins (Keverne et al. 1978), squirrel monkeys (Caine and Marra 1988), and preschool children (La Freniere and Charlesworth 1983) (but see also Torres de Assumpção and Deag 1979). This relation between dominance and social vigilance suggests that prevention of aggressive encounters is an important function of within-group vigilance. Thus, social vigilance may be used by subordinates to proactively seek information on the whereabouts of potential aggressors, which then allows for distance regulation towards these individuals (Chance 1967; Rowell and Olson 1983; Caine and Marra 1988; Alberts 1994; McNelis and Boatright-Horowitz 1998; Watts 1998; Blois-Heulin 1999; Treves 2000). This urge to detect, avoid and remain informed about potential aggressors is considered especially important in aggressive, intolerant species and/or species that lack the ability of communicating formal submission signals, such as patas monkeys (Rowell and Olson 1983; Caine and Marra 1988; Isbell and Pruetz 1998; Thierry et al. 2008; see also Evers et al. 2011 for more references).

In this context of aggressor avoidance, in line with Chance (1967) we can distinguish between two different forms of social vigilance: First, an individual may selectively monitor a particular, potentially dangerous group member by remaining visually oriented towards this animal to keep track of its further actions. Second, individuals may actively scan the whole social environment for nearby potential attackers. In this way, potential aggressors are detected in time and can then be avoided. Both monitoring and scanning behavior have been observed in several primate species, e.g. in macaques (Pitcairn 1976; Haude et al. 1976; Deaner et al. 2005), baboons (Alberts 1994), capuchins (Pannozzo et al. 2007), patas monkeys (Rowell and Olson 1983; McNelis and Boatright-Horowitz 1998), talapoins (Keverne et al. 1978), squirrel monkeys (Caine and Marra 1988), preschool children (La Freniere and Charlesworth 1983), mangabeys (Blois-Heulin 1999; Blois-Heulin and Girona 1999) and gorillas (Watts 1998). Note that authors of previous studies on primate social vigilance have used various definitions and terms for scanning and monitoring. Throughout this paper, we will utilize the definitions given above.

Individual variation in social vigilance has been suggested to result in dominants ending up at the “center of attention”, as well as at the spatial center of the group (Chance 1967; Chance and Jolly 1970). Chance (1967) proposed that “spatial features [of a primate group] are the outcome of subordinates behavior and of the attention to the dominant animal.” Unfortunately, these authors did not formulate a specific (testable) hypothesis about how exactly social vigilance may contribute to the spatial group structure. Nevertheless, there is some evidence that this relationship may exist (Table 1): in many primate species, where social vigilance is dominance-related, also a central-peripheral spatial group structure, with central dominants and more peripheral subordinates, is found. However, this link is weak, since it concerns only a concurring of described features. To our knowledge, no direct evidence for a causal link between social vigilance and socio-spatial group structure is available. Thus, it is yet unclear how this interrelationship may come about. Social vigilance, a socio-cognitive feature, crucial for distance regulation within the group, has also not yet been studied in simulation models. Based on the suggestions by Chance (1967) and on what has since then become known about primate social vigilance, we hypothesize that a central-peripheral group structure is reinforced by and arises from dominance related variation in social vigilance. More specifically, we hypothesize that individual variation in monitoring and scanning behavior causes spatial centrality of dominants. To find out how exactly this group pattern will arise from individual variation in monitoring and scanning behavior we implemented these behaviors in a simulation model, allowing us to test our hypothesis.

**Table 1:** Overview of several primate species and the specific group properties that have been reported for them

Species	Dominance-related social vigilance structure	Dominance-related spatial structure
*Macaca mulatta*	Haude et al. (1976); Deaner et al. (2005)	Southwick et al. (1965); Kaufmann (1967)
*Macaca fuscata*		Itani (1954); Imanishi (1960); Yamada (1966); Sugiyama and Ohsawa (1982); Wada and Matsuzawa 1986
*Macaca nemestrina*		Jensen and Tokuda (1974)
*Macaca arctoides*		Lopez-Lujan et al. (1989); Rasmussen and Farrington (1994)
*Papio cynocephalus*	Alberts (1994)	Washburn and DeVore (1961); Hall and DeVore (1965)
*Papio ursinus*		Busse (1984)
*Cebus apella*	Pannozzo et al. (2007)	Janson (1990)
*Cebus nigrivittatus*		Robinson (1981)
*Cebus capucinus*		Hall and Fedigan (1997)
*Erythrocebus patas*	McNelis and Boatright-Horowitz (1998)	
*Miopithecus talapoin*	Keverne et al. (1978)	
*Saimiri scireus*	Caine and Marra (1988)	
*Homo sapiens*	La Freniere and Charlesworth (1983)	

To investigate the mutual links between individual properties and group-level patterns (Hinde 1976), we use agent-based models. Agent-based models (ABMs, also called individual-based models or IBMs) are a well-established tool to systematically study and understand structuring mechanisms in a complex system of moving and interacting entities (Hogeweg and Hesper 1979; Bryson et al. 2007). Using a spatially explicit and individual-oriented model formalism allowed us to investigate how individual variation in behavioral, social and spatial properties may relate to each other. ABMs have been studied to understand how socio-spatial group patterns may emrge from interactions between individuals in primates and other species (Hemelrijk 2000; Bryson et al. 2007; Sellers et al. 2007; Rands et al. 2008; Sueur et al. 2010; see also Evers et al. 2011 for additional references). For instance, several ABM studies have demonstrated that spatial centrality of dominants may emerge in the absence of predation and spatial preferences, simply as a side effect of dominance relations and resulting differential movement (Hemelrijk 1998; Evers et al. 2011). This offered a parsimonious alternative to Hamilton’s “selfish herd theory” (1971).

In the current study, we set out to explore if and how variation in social vigilance may affect socio-spatial patterns in primate groups. Since monitoring and scanning may primarily serve more effective aggressor avoidance, we investigated both in this specific context. We first constructed a model of a group of primates, where individuals employ grouping behavior, dominance interactions and spatial avoidance of potential aggressors (cf. the *avoidance model* in Evers et al. 2011). We contrast this *avoidance model* which lacks any social vigilance behavior with two models that each additionally include one of the two types of social vigilance, reported in the primate literature: a) monitoring a specific potential aggressor to remain informed on its whereabouts (*monitoring model*) or b) scanning the whole group to detect potential aggressors (*scanning model*). In these three models, individual variation in fleeing tendency and avoidance tendency is present, which is known to affect the socio-spatial group structure (Hemelrijk 1998; Evers et al. 2011). Therefore, to assess the isolated effect of social vigilance (specifically scanning), we implemented a fourth model (*scanning control model*), where we eliminated the structuring effect of individual variation in fleeing and avoidance. This way we were able to test whether individual variation in social vigilance (i.c. scanning) alone is already sufficient to generate a central-peripheral group pattern.

## Methods

Simulations were run using NetLogo 4.0.3 (Wilenski 2007). The program code of all models is available on the website of the first author (http://web.science.uu.nl/behaviour/Evers/index.html). Definitions and values of the model parameters can be found in Table 2. Below, we describe our models according to the updated ODD protocol (Grimm et al. 2010).

**Table 2:** Parameters, definitions and values of the avoidance, monitoring and scanning model

Parameter	Description	Value
General parameters		
D	Grid unit	1 m
T	Time step	1 s
FIELD_SIZE	Field size	300 × 300 m
N	Number of individuals in group	30
PERS_DIST	Maximum distance, within which others can be encountered	4 m
NEAR_DIST	Maximum preferred distance to the group	20 m
MAX_DIST	Maximum distance monkeys are able to see	50 m
FAR_DIST	Maximum preferred distance to the furthest group member	${\text{NEAR}}\_{\text{DIST}}*\sqrt {N} \approx {11}0 {\text{m}}$
MIN_OTHERS	Minimum preferred number of conspecifics within NEAR_DIST	3
MAX_DOM	Maximum dominance strength	1.0
myDOM_i_	Dominance strength of individual i	(i * MAX_DOM) / N
VIEW_ANGLE	Default view angle	120°
ChaseD	Distance the winner of a fight chases the loser	1 m
FleeD	Distance the loser of a fight flees from winner	2 m
WalkD	Default distance an individual walks	1 m
Avoidance parameters		
AvoidD	Distance an individual moves away from avoidee	2 m
AV_DOM_DIFF	Avoidance dominance difference; difference in strength above which an agent is considered a potential aggressor and consequently avoided	0.4
AV_DIST	Avoidance distance; spatial distance within which potential aggressors are avoided	15 m
Scanning parameters		
MAX_ANGLE	View angle when scanning	360°
myVIEW_ANGLE	View angle employed by individual i, depending on whether i employs scanning at this moment	VIEW_ANGLE or MAX_ANGLE
P (scan_i_)	Scanning tendency of individual i	(MAX_DOM/2N)+MAX_DOM-myDOM_i_

### Purpose

The models described in this paper serve two main purposes. First, we wanted to assess whether and how individual variation in primate social vigilance (monitoring or scanning) can affect or enhance certain properties at the group level, such as spatial centrality of dominants and relative encounter rates. Second, we wanted to explore whether and how individual variation in social vigilance alone may already be sufficient to result in spatial centrality of dominants, a pattern that has been reported for several primate species. The experimental set-up is explained in the section Simulation experiments and summarized in Table 3.

**Table 3:** Experimental set-up and characteristics of the compared models

Factor		Fleeing frequency	Avoidance tendency	Monitoring tendency	Scanning tendency
1.	Avoidance model	Variable	Variable	Not employed	Not employed
2.	Monitoring model	Variable	Variable	Variable	Not employed
3.	Scanning model	Variable	Variable	Not employed	Variable
4.	Scanning control model	Equal	Not employed	Not employed	Variable

### Entities, state variables and scales

We model the interactions and movements of 30 individuals. These individuals are characterized by their dominance strength (myDOM), which ranges from *MAX*_*DOM / N* (for the lowest-ranking individual) to *MAX*_*DOM* (highest-ranking). Dominance strength does not change in time or after interactions (cf. the model in the appendix of Bryson et al. 2007).

When scanning behavior is included in the model*,* individuals are also characterized by their scanning tendency, which is inversely related to dominance strength and thus also constant over time.

Furthermore, individuals are characterized by their spatial coordinates, these may change during the whole simulation run.

The modeled environment is a continuous two-dimensional grid (300 × 300 grid units) with a torus shape to exclude disturbing border effects. One grid unit resembles 1 “meter”. We did not explicitly implement ecological features of the environment; in the model an individual’s environment is purely social. This also implies that the model individuals do not engage in foraging behavior. Thus, we model a group that is not traveling.

One time step in the simulation resembles 1 “second” and simulations were run for 72,000 time steps (resembling 20 observation “hours”).

### Process overview and scheduling

Our model is event-driven. During a simulation run, individuals’ activations are regulated by a timing regime. Agents are activated in a cyclic, asynchronous way. Each time, the agent with the lowest schedule time is activated first. After activation, this agent’s next activation is scheduled. The remaining time until its next scheduled activation is randomly drawn from a negative exponential distribution with a mean of 10 time steps. In other words, events are randomly distributed in time. Scheduled times are on a continuous range. If an action involves other individuals as well, each participant gets scheduled anew for its next action.

**Fig. 1:**
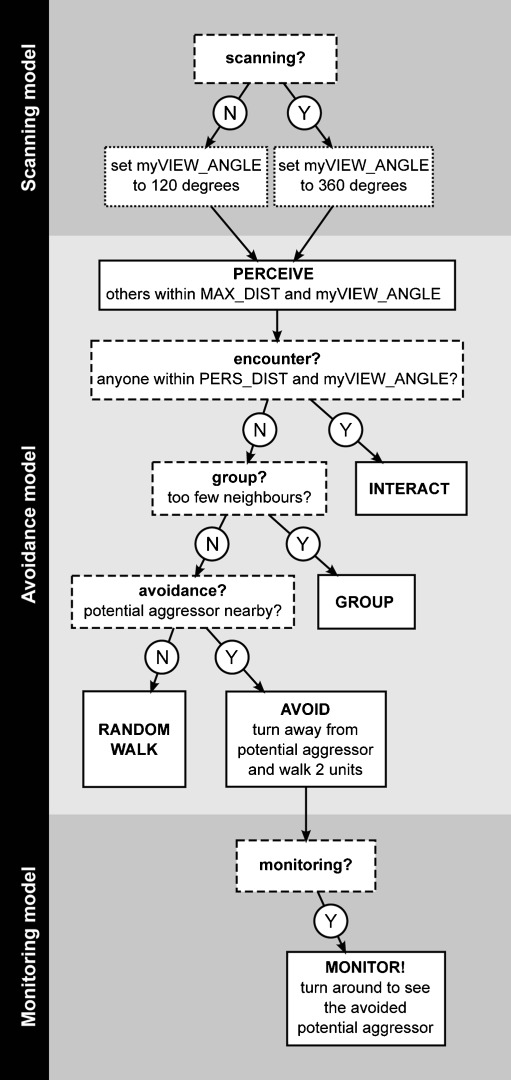
Interaction rules. Model individuals execute a hierarchical behavioral script. The script is starting at the top and ending in one of four or five (depending on the model) possible end states

On activation, individuals execute an action-selection protocol (Fig. 1). This protocol goes through a number of decisions to produce the behavior appropriate to the social situation. The decision procedure is structured hierarchically: interactions have priority over grouping, grouping has priority over avoidance and avoidance has priority over moving within the group.

The action-selection protocol starts with ego perceiving the configuration of the social environment. Right upon perception, ego checks whether another individual is encountered, which will lead to an agonistic interaction (see submodel Agonistic interactions below). If no one was encountered, ego turns and moves towards the group if necessary (see submodel Grouping below). If grouping is not necessary, ego may chose to avoid potential aggressors at a distance (see submodel Avoidance below). If none of the above actions were selected, ego simply moves randomly through the group (see design concept Stochasticity below).

When monitoring behavior was included in the model, avoidance of a specific individual was immediately followed by monitoring this individual (see submodel Monitoring below). When scanning behavior was included in the model, the action-selection protocol was preceded by, and thus started with the decision of ego, whether to employ scanning behavior or not (see submodel Scanning below).

### Design concepts

#### Emergence

In the models, individuals have a preference to stay near the group. However, individuals do not have any preference for a specific spatial location within the group (e.g. the group center). Any structure or pattern in the spatial configuration of the group is thus not imposed by the model rules, but arises purely from the interactions between the individuals and the resulting movements.

Which individuals will encounter each other regularly (i.e. the encounter structure) will arise as a result of the emergent spatial structure in combination with the behavioral rules of the specific model.

#### Sensing

**Fig. 2:**
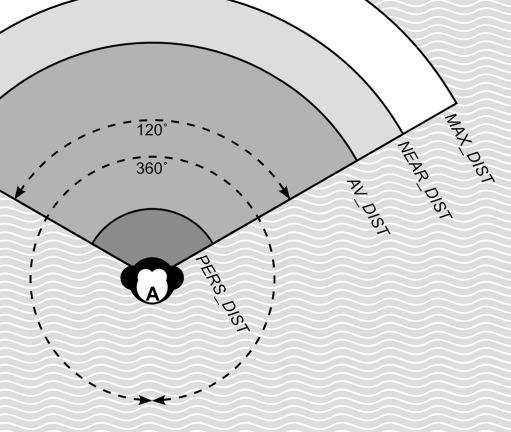
Perception. Model individuals perceive other group members within a default view angle of 120º. When scanning, individuals perceive others within an angle of 360º. The maximum distance within which another can be seen is MAX_DIST. Distances in the Figure are not to scale

Individuals in the models are capable of perceiving the spatial distance and the dominance strength of others that are dwelling within a view angle of 120º and a maximum perceivable distance of 50 m (VIEW_ANGLE and MAX_DIST in Fig. 2). Note that when scanning behavior is included in the model, whenever an individual is scanning, its view angle is 360º (see submodel Scanning below).

#### Interaction

Social interactions between individuals in the model may take place in several ways. Group members that were perceived within PERS_DIST may be chosen by ego as agonistic interaction partner (see submodel Agonistic interactions for more details). An agonistic interaction may then result in distance regulation by the lower-ranking individual, i.e. subordinate moving away from the dominant. If the dominance interaction escalates into a fight, the winner of the fight may chase the loser.

Furthermore, potential aggressors that were perceived within AV_DIST may be avoided. Group members that were perceived within MAX_DIST may be approached by ego when grouping.

#### Stochasticity

In our model, when two individuals engage in a fight, the winner is stochastically determined: individual A wins from B, depending on its win chance, *w*_*AB*_ (cf. Evers et al. 2011). A higher difference in dominance strength results in a higher win chance for the dominant individual.

When executing a random walk, individuals simply move forward (for WalkD = 1 m) and with a chance of 0.5, they then turn randomly up to 180º to the right or left.

In the scanning model, individuals may employ scanning. Prior to each activation, ego decides whether to employ scanning during the next action-selection cycle, depending on its scanning tendency.

#### Observation

To assess socio-spatial group properties within each model, we used several measures. We recorded each individual’s distance to the centroid of the group (cf. Evers et al. 2011).

To calculate spatial group spread, we recorded the furthest neighbor distance within the group (the distance between the two individuals in the group that are furthest away from each other).

We assessed how dyadic distances and the number of encounters were distributed among all possible dyads. The spatial dyadic distances were simply recorded over time. To measure the total number and direction of encounters per simulation run (the encounter structure), we recorded the identity of the group members that ego had selected as interaction partner.

We recorded how perception was distributed and directed among group members (the perception structure). For each individual, we scored which other individuals it perceived within MAX_DIST and its employed view angle at the time of measuring. This was recorded several times per run (see below), using the “one–zero” sampling technique. Thus per dyad, possible scores were 1 (perceived) or 0 (not perceived) per sample. Note that depending on whether an individual was scanning or not, its employed view angle was either 360 or 120º.

We only recorded data during the last 10 “hours” of each simulation run, to avoid transient spatial and social group effects due to the initial random placement. All measures of the socio-spatial structure of the group (distance to centroid of the group, spatial group spread, dyadic distances, encounter structure and perception structure) were recorded every 900 time steps, which was equivalent to 15 “minutes”. All measures, except the number of encounters per dyad, were averaged over time for each simulation run. For the number of encounters per dyad all occurrences were recorded per simulation run. Per model, 50 independent simulations were run.

#### Initialization

At the initialization of each simulation run, the x-coordinates and the y-coordinates of all individuals are drawn from a normal distribution around an arbitrarily chosen position on the spatial grid (standard deviation = 10 grid units), independent of an individual’s dominance strength. Their initial heading was set to a random number between 1 and 360º. Furthermore, the initial schedule time for each individual is randomly drawn from a negative exponential distribution with a mean of 10 time steps. Lastly, individuals get assigned their dominance strength (myDOM), which ranges from *MAX*_*DOM / N* (for the lowest-ranking individual) to *MAX*_*DOM* (highest-ranking) and which stays constant over the course of the simulation.

### Submodels

This section describes each process that is executed by the model entities in more detail. Moreover, how each process is modeled and parameterized is explained.

#### Movement

Movement of the model individuals may either be motivated by explicit social factors, such as grouping, fleeing, chasing or avoidance, where movement is directed away from or towards one specific individual, or is else implemented as a random walk of WalkD = 1 m (see also design concept: Stochasticity).

Parameter values concerning the random walk were kept the same as in our earlier model (*avoidance model* cf. Evers et al. 2011), where WalkD was adapted from the DomWorld model by Hemelrijk (1998, 2000), while values for the other parameters were chosen arbitrarily.

#### Grouping

To stay relatively close to group members, ego checks whether at least three group members (MIN_OTHERS) are situated within a distance of 20 m (NEAR_DIST) within its employed view angle. If not enough group members were perceived, ego tries to find another group member within the maximum distance they can see (MAX_DIST = 50 m), or else within a broader view angle (360º) by looking around (Fig. 2). Of the perceived individuals, one is selected randomly and approached by 1 m (WalkD). Additionally, individuals are not allowed to increase the distance towards the furthest individual more than a certain distance (FAR_DIST). This procedure ensures a coherent group, which does not split up (see Evers et al. 2011).

Parameters concerning the grouping behavior of the model individuals were kept the same as in our earlier model (*avoidance model* cf. Evers et al. 2011), where MAX_DIST, VIEW_ANGLE and WalkD were adapted from the DomWorld model by Hemelrijk (1998, 2000), NEAR_DIST was adapted from an earlier reimplementation of the DomWorld model (Bryson et al. 2007) and MIN_OTHERS was adapted from van der Post et al. (2009).

#### Agonistic interactions

When one or more group members are perceived within a personal distance of 4 m, ego chooses the nearest individual as an interaction partner. In our models interactions are always dyadic. For each interaction, this partner choice is recorded and scored as an encounter. Thus, encounters are directed from one individual (ego, who perceived the other first) to another (chosen partner).

Ego may either challenge its interaction partner or flee from it for 2 m. This decision depends on the chance of winning a fight with the opponent (de Vries 2009; Evers et al. 2011). As a response to a challenge, the opponent may either reject or agree to engage in a fight, depending on its own expected win chance (cf. Evers et al. 2011). If one of the opponents declines and flees away, the conflict is settled. Only if both individuals agree to a fight does an actual fight take place. The winner of a fight is stochastically determined and depends on both individuals’ win chance. Subsequent to a fight, the loser flees from the winner for 2 m (FleeD), while the winner chases the loser by running after him for 1 m (ChaseD).

The agonistic interaction procedure described above results in low-ranking individuals losing and fleeing more often than high-ranking ones. When controlling for individual differences in fleeing rate we simply assigned a win chance of 0.5 to each individual, independent of its actual dominance strength. In this way, fleeing rates were equal among individuals, while e.g. scanning frequency did still differ.

Parameters concerning the dominance interactions of the model individuals were kept the same as in our earlier model (*avoidance model* cf. Evers et al. 2011), where PERS_DIST, ChaseD and FleeD were adapted from the DomWorld model by Hemelrijk (1998, 2000).

#### Avoidance

The model individuals are capable of avoiding potential aggressors at a distance. Whether ego may avoid another individual depends on the difference between both individuals’ dominance strength and on the spatial distance to that specific animal. Whenever other group members have a larger dominance strength than the sum of ego’s dominance strength and AV_DOM_DIFF and whenever such individuals (potential aggressors) are closer to ego than AV_DIST, ego may avoid them. If these conditions are true for several group members, ego chooses to avoid the nearest of these individuals. Therefore, by definition avoidance behavior is more frequently employed by lower-ranking individuals.

The actual avoidance behavior is implemented in the following way. If potential aggressors are detected, the nearest one is selected and avoided: ego turns away from this individual (180º) and walks away for 2 m.

Avoidance can either be a stand-alone reactive action on the perception of a potential aggressor, or it can be accompanied by additional mechanisms to prevent close proximity to potential aggressors in the first place by employing proactive (in contrast to reactive) detection of potential aggressors, namely monitoring and scanning.

Note that extreme conditions for aggressor avoidance (namely avoidance of many dominants even when they were still at a large distance, i.e. small AV_DOM_DIFF and large AV_DIST) have been shown to result in subgroup formation in our model, even within the maximum allowed group spread (FAR_DIST) (Evers et al. 2011). In the current paper, we chose conditions (intermediate AV_DOM_DIFF and AV_DIST) that resulted in coherent groups that lacked any subgroup formation.

#### Monitoring

Individuals may employ monitoring behavior to stay informed about the actions of a particular potential aggressor. Monitoring behavior is exclusively directed at potential aggressors, i.e. the few highest-ranking individuals, and is only executed in conjunction with avoidance behavior. Avoiding a potential aggressor, i.e. moving away from it, results in a larger distance to the avoided individual and thus in a smaller aggression risk. Monitoring is employed right after detection and avoidance of a potential aggressor. From a larger distance, ego turns around towards the potential aggressor, to check whether it is now at a large enough distance (AV_DIST) from this specific individual or whether avoidance should be employed once more. As monitoring is in our model connected to avoidance behavior, it is (just like avoidance behavior itself) inversely related to dominance strength: low-ranking individuals frequently employ monitoring behavior.

#### Scanning

Individuals may frequently scan their whole surrounding environment (within MAX_DIST) to detect known potential aggressors. When employing scanning behavior, an individual is turning its head right and left, thus expanding its view angle to 360º (instead of the default view angle of 120º). Individuals with low dominance strength (myDOM) have a higher chance to employ scanning. Scanning tendency is thus inversely related to dominance strength and is calculated as follows (for individual A):

$$ P\left( {sca{{n}_A}} \right) = \frac{{MAX\_DOM}}{{2N}} + MAX\_DOM - myDO{{M}_A}, $$

where MAX_DOM is 1.0 and N is the group size. Prior to the behavioral script, ego decides whether to employ scanning, during the next action-selection cycle. When scanning, also individuals outside of the default view angle (120º) may be detected and avoided. As scanning for potential aggressors occurs indivisibly with scanning for conspecifics in general, the additional social information perceived through scanning is not exclusively used for avoidance behavior, but is also used when employing grouping behavior or when encountering others.

### Simulation experiments

To investigate the implications of monitoring and scanning on the socio-spatial properties of primate groups, we compared a basic model where individuals avoid potential aggressors, but do not employ monitoring or scanning (*avoidance model*), to two models that additionally include either monitoring (*monitoring model*) or scanning (*scanning model*). As basic model, we used the *avoidance model* with parameter settings that result in a coherent group that does not split up (Evers et al. 2011). In this *avoidance model*, individuals follow a set of rules, inspired by real primates: (1) Individuals prefer to have at least three other group members in sight. (2) Individuals avoid potentially aggressive group members. (3) Individuals move within the group. (4) Individuals may engage in agonistic interactions.

In the *monitoring model*, individuals follow the same rules as the *avoidance model*, yet additionally individuals employ monitoring behavior. Thus, after detection and avoidance of a specific potential aggressor, the avoider may monitor this specific aggressor to stay informed about its further actions.

In the *scanning model*, individuals follow the same rules as in the *avoidance model*, yet additionally individuals employ scanning behavior. Thus, individuals may regularly scan their entire social environment to detect potential aggressors more efficiently.

Individual variation in social vigilance may also arise in contexts other than aggressor avoidance (e.g. due to maternal care or mate guarding). To check whether individual variation in social vigilance alone may be sufficient to generate socio-spatial group patterns, we investigated the properties of an additional model, where individuals merely differ in their scanning tendency (*scanning control model*). In this model, individuals do not differ in their fleeing frequency and do not employ any avoidance. Thus, in the *scanning control model*, the structuring effect of avoidance and variation in fleeing frequency (as found in Evers et al. 2011) was removed.

Note that the isolated effect of monitoring has not been assessed, because employing monitoring behavior without avoidance behavior is not possible in our model: avoiding a potential aggressor implies orienting (and moving) away from it. Subsequent monitoring enables the individual to visually orient back towards the potential aggressor. However, when no avoidance has been employed (yet), the individual would still be oriented towards the perceived potential aggressor.

### Statistical analysis

To measure how individual differences in monitoring tendency and scanning frequency were related to the individuals’ spatial position within the group, a regression line was fit to assess the relation between an individual’s centrality (distance to the centroid of the group) and the individual’s dominance strength (myDOM) per simulation run. The steeper this regression line, the more pronounced the relation between centrality and dominance strength was. A Wilcoxon rank sum test with continuity correction was used to assess whether monitoring or scanning affected the relation between centrality and dominance strength, i.e. the slope of the regression line. The two extended models (*monitoring model* and *scanning model)* were compared to the basic model (*avoidance model)*.

A *t* test was used to demonstrate a relation between spatial centrality and dominance strength in the *scanning control model.* The slope of the regression line in the *scanning control model* was compared to zero. Normality assumptions were checked using Shapiro-Wilk tests.

Statistical analyses were performed in R 2.10.1 (R development Core Team, Vienna, Austria).

## Results

### Avoidance

**Fig. 3:**
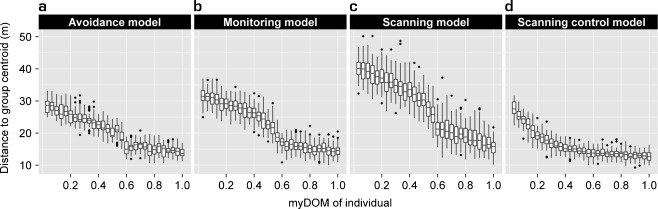
Centrality of dominants. This graph shows the relationship between an individual’s dominance strength (myDOM) and its centrality (distance to the centroid of the group in meters) for different models. **a** *Avoidance model*. **b** *Monitoring model*. **c** *Scanning model*. **d** *Scanning control model*. Small distances to the arithmetic group center indicate more central positions. When the relation between dominance strength and centrality is steeper (**b** and **c**), centrality of dominants is more pronounced. Depending on the model, a low dominance strength further implies low win chance and thus frequently employed fleeing behavior (*avoidance*, *monitoring* and *scanning model*), frequently employed avoidance behavior (*avoidance*, *monitoring* and *scanning model*), frequently employed monitoring behavior (*monitoring model*) and frequently employed scanning behavior (*scanning* and *scanning control model*). Boxplots show 50 simulation runs, averaged over time

**Fig. 4:**
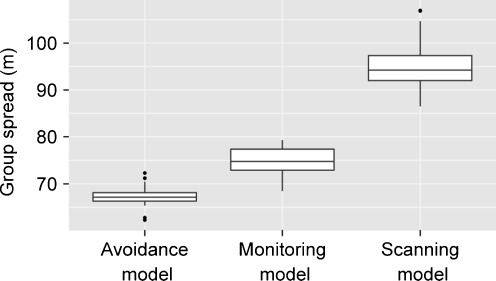
Group spread. This graph shows the groups spread (spatial group diameter in meters) for the different models *avoidance model*, *monitoring model* and *scanning model*. Boxplots show values of 50 simulation runs, averaged over time

**Fig. 5:**
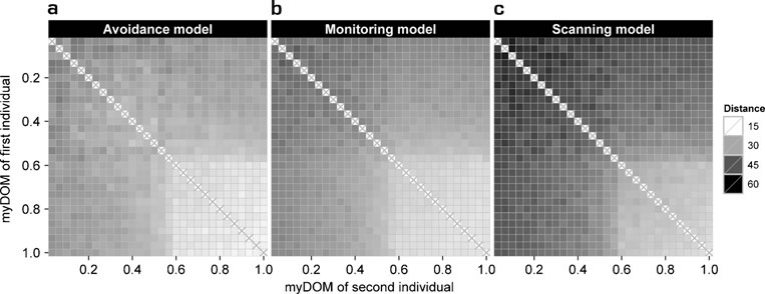
Spatial structure. This graph shows the distribution of dyadic distances (in meters) among the individuals of a group for different models. **a** *Avoidance model*. **b** *Monitoring model*. **c** *Scanning model*. The x-axis shows the dominance strength of the first individual and the y-axis the dominance strength of the second individual per dyad. For further implications of an individual’s dominance strength depending on the model, see the Fig. 3 legend. Plots show the mean values of 50 simulation runs, averaged over time. Darker shades represent larger dyadic distances. Values at the diagonal (x) are by default not applicable. Note that the distance matrices are by definition symmetrical

**Fig. 6:**
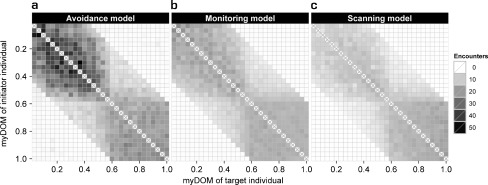
Encounter structure. This figure shows the distribution and direction of encounters among the individuals of a group for different models. **a** *Avoidance model*. **b** *Monitoring model*. **c** *Scanning model*. Encounters are directed from initiators (y-axis) to targets (x-axis), both are ordered by dominance strength (myDOM). For further implications of an individual’s dominance strength depending on the model, see the Fig. 3 legend. Plots show the mean values of 50 simulation runs. Dark shades represent frequent encounters. Values at the diagonal (x) are by default not applicable

**Fig. 7:**
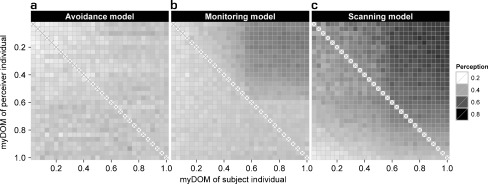
Perception structure. This figure shows the distribution and direction of perception among the individuals of a group for the different models. **a** *Avoidance model*. **b** *Monitoring model*. **c** *Scanning model*. Perception is directed from perceivers (y-axis) to subjects (x-axis), both are ordered by dominance strength (myDOM). For further implications of an individual’s dominance strength depending on the model, see the Fig. 3 legend. Plots show the mean values of 50 simulation runs. Dark shades represent frequent perception. Values at the diagonal (x) are by default not applicable

A detailed description and examination of the properties of the *avoidance model* can be found elsewhere (Evers et al. 2011). For the parameter settings chosen here, variation in fleeing frequency combined with variation in avoidance behavior (both due to differences in dominance strength) resulted in a clear central-peripheral group pattern (Fig. 3a): subordinates (avoiders) populated the group periphery, as they frequently avoided and fled from central dominants (avoidees). Avoidance behavior resulted in fairly spread out groups (Fig. 4): the average furthest neighbor distance was 67.4 ± 1.9 m (mean ± standard deviation, *N* = 50 simulation runs). Dyadic distances among dominants (avoidees) were smaller compared to distances among subordinates (avoiders) and compared to distances between subordinates (avoiders) and dominants (avoidees) (Fig. 5a). More encounters took place among individuals of the same subgroup, most of them among avoiders (Fig. 6a). We also measured who is perceived by whom how often. In the *avoidance model* there was no noticeable variation within this perception structure and perception was almost equally distributed among all individuals (Fig. 7a).

### Monitoring

The central-peripheral structure in the *monitoring model* was slightly more pronounced compared to the *avoidance model*: individual differences in the distance to the centroid of the group were higher in the *monitoring model* (Fig. 3b). This was also apparent in the slope of the regression line, which was significantly steeper in the monitoring model than in the avoidance model (Wilcoxon rank sum test: *W* = 2438, *P* < 0.001; mean slopes ± standard deviation = −0.71 ± 0.056 and −0.55 ± 0.050, respectively). Furthermore, groups in the *monitoring model* were significantly more spread out compared to groups in the *avoidance model* (Wilcoxon rank sum test: *W* = 21, *P* < 0.001; mean average furthest neighbor distance ± standard deviation = 75.0 ± 2.6 m and 67.4 ± 1.9 m, respectively; Fig. 4). This can be explained as follows. By employing monitoring, individuals remained informed on their distance towards central avoidees. This allowed them to constantly adjust their spatial distance towards these animals. More frequently employed avoidance behavior then resulted in more spread out groups.

Higher group spread in the *monitoring model* was also reflected in the dyadic spatial distances among group members (Fig. 5b). Dyadic distances increased not only between monitoring subordinates and central dominants, but, as a side effect, also among subordinates.

In the *monitoring model*, encounters among individuals were less frequent than in the *avoidance model*, especially among subordinates (Fig. 6b). However, spatial distances among subordinates only partly explain the decrease in encounters. An additional underlying mechanism concerns the visual orientation of subordinates. Subordinates frequently oriented towards central dominants in the *monitoring model* (Fig. 7b). This “selective attention” towards dominants “distracted” subordinates from other peripheral subordinates and, thereby, lowered the chance of encountering them.

### Scanning

The central-peripheral structure in the *scanning model* was clearly more pronounced than in the *avoidance model*, since individual distances to the centroid of the group were most differentiated (Fig. 3c). This was also apparent in the slope of the regression line, which was significantly steeper in the scanning model than in the avoidance model (Wilcoxon rank sum test: *W* = 2500, *P* < 0.001; mean slopes ± standard deviation = −0.95 ± 0.059 and −0.55 ± 0.050, respectively). Therefore, individual variation in scanning tendency reinforced the already existing spatial structure, which emerged from individual variation in fleeing frequency and avoidance behavior (Evers et al. 2011).

This raises the question whether individual variation in scanning tendency alone may already generate a central-peripheral structure. To test this, we implemented the *scanning control model*, in which we excluded the structuring effects of variation in fleeing frequency and avoidance behavior by setting all win chances to 0.5 and by excluding avoidance behavior. Surprisingly, a central-peripheral group structure still emerged (Fig. 3d) and the slope of the regression line was significantly different from zero (t test: *t* = −94.558, *P* < 0.001; mean slope ± standard deviation = −0.40 ± 0.030). This came about as follows. Frequently scanning individuals moved further away from the group, because they could still perceive enough group members, even when being oriented away from the group. In contrast, rarely scanning individuals had to turn around, and thus moved back to the group immediately, when approaching the periphery resulted in too few perceived group members. Differential perception resulted in differential grouping behavior and thereby in a central-peripheral group structure with frequent scanners at the periphery and infrequent scanners at the group center. We do not describe any further characteristics of the *scanning control model*, as this model only served to show the structuring effect of individual variation in scanning tendency.

In the *scanning model*, groups were significantly more spread out compared to the *avoidance model* (Wilcoxon rank sum test: *W* = 0, *P* < 0.001; mean average furthest neighbor distance ± standard deviation = 95.0 ± 4.6 m and 67.4 ± 1.9 m, respectively; Fig. 4). There are two ways to explain this pattern. First, subordinate individuals were occupying peripheral positions in the group. Frequent scanning allowed these individuals to move even further away from the group, while still perceiving the required number of other group members. Second, frequently scanning the group allowed individuals to avoid potential aggressors (central dominant individuals) more often. More frequent avoidance caused subordinates to move further away from central aggressors and resulted in higher group spread.

That groups were more spread out in the *scanning model* was also reflected in the dyadic distances among the group members (Fig. 5c). Especially distances among frequently scanning subordinates and between subordinates and dominants were increased relative to the *avoidance model*.

In the *scanning model*, encounters among individuals were less frequent than in the *avoidance model*, especially among subordinates (Fig. 6c). Frequent scanning of subordinates enabled enhanced perception of other group members (Fig. 7c). As a result, a higher encounter rate for subordinates would be expected, as they may also encounter individuals outside of the default view angle of 120º. However, the perceptive “advantage” of scanning was counteracted by the large dyadic distances and ultimately resulted in decreased encounter rates.

## Discussion

We presented a number of models, investigating how individual variation in social vigilance (monitoring or scanning) in the context of spatial avoidance of aggressors affect group-level properties (the socio-spatial structure). Our results showed that individual variation in social vigilance resulted in more spread out groups, decreased encounter rates between subordinates and reinforced the existing central-peripheral spatial structure of the group. Moreover, such a central-peripheral group structure may also emerge from individual variation in scanning tendency alone.

### Emergence of a central-peripheral group structure

In the basic *avoidance model*, a central-peripheral group structure emerged from individual variation in fleeing and avoidance behavior (Evers et al. 2011). When individual variation in monitoring or scanning was added to the model, this spatial structure was reinforced by monitoring and, to a higher degree, by scanning behavior. The last result suggested that, besides reinforcing an existent group-structure, individual variation in social vigilance alone may even be sufficient to result in a central-peripheral group structure. This was explored in the *scanning control model*, which did not include any dominance-related variation in avoidance or fleeing rates. The individual variation in scanning tendency within the group indeed was sufficient to generate a central-peripheral group pattern. Frequently scanning individuals occupied more peripheral spatial positions in the group, even when the other structuring factors (variation in fleeing frequency and avoidance behavior) were excluded. Chance (1967) suggested that variation in frequency and direction of social vigilance might put dominant individuals into the spatial center of the group, but to our knowledge this has never been measured or tested. Our model shows indeed such a spatial pattern and explains how this pattern may arise purely as a side effect of individual differences in social vigilance. In contrast to the less frequently scanning dominants, frequently scanning subordinates often perceived group members outside of the default view angle. Therefore, frequent scanning allowed subordinates to employ grouping behavior less often compared to dominants. As a consequence, scanners occupied more peripheral positions. Of course, our results are relying on a number of specific assumptions we made in the model (e.g. the grouping rules), and the validity of these assumptions remains to be investigated empirically. We believe that one of the main advantages of agent-based modeling is that it may help to discover plausible behavioral rules to be verified empirically. Thus, ABM allows for a strong interplay between constructing an explanatory model and obtaining empirical data (de Vries and Biesmeijer 1998).

In animals, vigilance is not only directed at conspecifics, but, more commonly, also at predators. Hamilton (1971) tried to link within-group variation in predator vigilance to spatial positions within the group. He hypothesized that the higher predation risk at the periphery of the group should result in more frequently employed vigilance at these spatial positions (“edge effect”). Since then, a correlation between higher degree of vigilance and peripheral positions within a group is usually attributed to higher predation risk at the group periphery, in primates (Robinson 1981; van Schaik and Noordwijk 1989), birds (Inglis and Lazarus 1981; Keys and Dugatkin 1990; Black et al. 1992) and other species (see Table 5.3 in Caro 2005). However, individual variation in vigilance may also originate from factors other than differential predation risk, for instance due to social factors, such as variation in risk of aggression (Chance and Jolly 1970), infanticide (Steenbeek et al. 1999) or competition for mates or resources (Caraco 1979; Cowlishaw 1998). Our *scanning control model* predicts that whenever variation in vigilance (in our case scanning frequency) is present in a group, frequently vigilant individuals may end up at the group periphery automatically, even in the absence of any predator or any other structuring factors such as aggressor avoidance or dominance interactions. Thus, when peripheral group members employ vigilance behavior more frequently, a premature conclusion about a possible adaptation to differential predation risk is best avoided. The actual underlying mechanism should be evaluated carefully per species and environment.

In a former study (Evers et al. 2011), we investigated how individual variation in movement properties may shape socio-spatial properties within a group. Three factors driving the emergence of a central-peripheral group structure have been identified, namely individual variation in fleeing frequency, avoidance behavior and average velocity (Evers et al. 2011). In the current paper, we identified another factor that may contribute to the centrality of dominants typically reported in primate studies: individual variation in scanning tendency. We conclude that individual variation in different types of behavior (fleeing, velocity, avoidance and scanning) can generate a central-peripheral spatial pattern in primate groups. High fleeing frequency, frequent avoidance behavior, high average velocity and high scanning tendency are all properties that are often found in subordinate members of primate groups (Chance 1956; Keverne et al. 1978; McNelis and Boatright-Horowitz 1998; Pannozzo et al. 2007; see Evers et al. 2011 for additional references). Disentangling this set of inter-related factors within a simulation model showed how each property independently may result in peripheral spatial positions of certain individuals within a group. We conclude that individual variation in different types of behavior (fleeing, velocity, avoidance and scanning) can generate a central-peripheral spatial pattern in primate groups. A central-peripheral group structure is, thus, a robust pattern, which may be driven by several independent mechanisms, commonly found in primate groups simultaneously.

### Spatial structure, encounter structure and social vigilance

While in all three models, the *avoidance*, *monitoring* and *scanning model*, a central-peripheral structure emerged; group spread was highest in the *monitoring* and *scanning model*. Interestingly, the causes of high group spread differed for the *monitoring* and the *scanning model*. Monitoring potential aggressors resulted in more effective avoidance behavior and therefore in more spread out groups. Scanning, however, not only allowed for more effective avoidance of potential aggressors, but also gave rise to less frequent grouping behavior as a side effect. Both of these effects caused the group in the *scanning model* to be the most spread out.

In the *monitoring* and in the *scanning model*, the encounter structure (the frequency and direction of encounters) was affected by both the spatial group structure and the variation in social vigilance. In the *monitoring model* the rate of encounters was decreased due to larger dyadic distances and additionally to the subordinates’ monitoring of central dominants. Subordinates mainly focused their vigilance on perceiving and avoiding dominants, thereby “losing sight” of other potential interaction partners (subordinates) at the periphery. In the *scanning model*, encounter frequencies among subordinates were also decreased due to large dyadic distances among peripheral individuals. Although scanning individuals had a higher chance to perceive and eventually encounter other group members, this was counteracted by the large inter-individual distances.

These results reveal that social group properties, such as encounter structure, are affected by, but not deducible from the average spatial distances alone. To reveal the underlying processes of socio-spatial group structure, spatial data of real primate groups are best analyzed in combination with data on social vigilance within a group.

### Further directions

Our model predicts that monitoring and scanning behavior reduces encounter rates and, therefore, aggression. It would be interesting to test our findings and compare encounter structures between species or groups that differ in the amount of social vigilance employed. In gorillas, females have been shown to attend differentially towards kin, mates or recent immigrants (Watts 1998). Similarly, chimpanzees adjust their level of social vigilance depending on their relationship quality with associates (Kutsukake 2006). Whether such variation in social vigilance is related to encounter rates could be investigated in differently composed groups.

Furthermore, in social groups monitoring behavior may provide cues to group members. Individuals may be able to use the direction of monitoring (the gaze direction of other individuals) to infer information about the location of potential aggressors and possibly also about the attentive and emotional state of the monitoring animal (Goossens et al. 2008; Teufel et al. 2010). Thus, while monitoring is used to obtain social information by the monitoring animal, the behavior itself may provide social information to others and thereby reinforce social group structure (Coussi-Korbel and Fragaszy 1995; Pannozzo et al. 2007). This opens up new research topics to investigate how social vigilance, and other ways of acquiring information from conspecifics (Bonnie and Earley 2007), may affect group-level properties in primates and other social animals.

In our model, social vigilance was directed at feared individuals. Of course, real primates may employ social attention also to detect preferred individuals, such as kin, mates or allies, and seek their proximity. For example, monitoring may help to stay informed about the spatial position of affiliates while moving into a different direction (a so-called “secondary referent” cf. Emory 1976; see also Virgo and Waterhouse 1969), and scanning might be used to quickly find and recruit potential coalition partners.

## Conclusion

By investigating the link between individual variation in social vigilance and socio-spatial group structure in an agent-based model, we offer a new perspective on the causal relations between different group-level patterns. Our model yields another possible explanation for one of the main questions in the primate literature: what causes centrality, in the light of a specific primate behavior: namely social vigilance. In line with the suggestion of Chance (1967), this agent-based model study provides a clear indication that variation in social vigilance may be an important structuring feature of primate social groups.
